# Serum metabolomic analysis reveals disorder of steroid hormone biosynthesis in patients with idiopathic inflammatory myopathy

**DOI:** 10.3389/fimmu.2023.1188257

**Published:** 2023-06-12

**Authors:** Tong Huo, Xueting Yuan, Jingyi Han, Jia Shi, Yuehan Xiong, Feng Tian, Zihan Xu, Menghua Cai, Yi Xu, Hui Chen, Xiaofeng Zeng, Wei He, Qian Wang, Jianmin Zhang

**Affiliations:** ^1^ Chinese Academy of Medical Sciences (CAMS) Key Laboratory for T Cell and Immunotherapy, State Key Laboratory of Medical Molecular Biology, Department of Immunology, Institute of Basic Medical Sciences, Chinese Academy of Medical Sciences and School of Basic Medicine, Peking Union Medical College, Beijing, China; ^2^ Department of Rheumatology and Clinical Immunology, Peking Union Medical College Hospital, Peking Union Medical College, Chinese Academy of Medical Sciences, Beijing, China; ^3^ National Clinical Research Center for Dermatologic and Immunologic Diseases, Ministry of Science & Technology, Beijing, China; ^4^ State Key Laboratory of Complex Severe and Rare Diseases, Peking Union Medical College Hospital, Beijing, China; ^5^ Key Laboratory of Rheumatology & Clinical Immunology, Ministry of Education, Beijing, China; ^6^ Department of Thoracic Surgery, Qilu Hospital of Shandong University, Jinan, Shandong, China; ^7^ Guidon Pharmaceutics, Beijing, China; ^8^ Haihe Laboratory of Cell Ecosystem, Chinese Academy of Medical Sciences and Peking Union Medical College, Tianjin, China; ^9^ Changzhou Xitaihu Institute for Frontier Technology of Cell Therapy, Changzhou, China

**Keywords:** idiopathic inflammatory myopathy (IIM), arachidonic acid metabolism pathway, steroid hormone biosynthesis pathway, serum metabolome, myositis-specific autoantibody

## Abstract

Idiopathic inflammatory myopathy (IIM) is a heterogeneous group of autoimmune diseases with various clinical manifestations, treatment responses, and prognoses. According to the clinical manifestations and presence of different myositis-specific autoantibodies (MSAs), IIM is classified into several major subgroups, including PM, DM, IBM, ASS, IMNM, and CADM. However, the pathogenic mechanisms of these subgroups remain unclear and need to be investigated. Here, we applied MALDI-TOF-MS to examine the serum metabolome of 144 patients with IIM and analyze differentially expressed metabolites among IIM subgroups or MSA groups. The results showed that the DM subgroup had lower activation of the steroid hormone biosynthesis pathway, while the non-MDA5 MSA group had higher activation of the arachidonic acid metabolism pathway. Our study may provide some insights into the heterogeneous mechanisms of IIM subgroups, potential biomarkers, and management of IIM.

## Introduction

Idiopathic inflammatory myopathy (IIM), also known as myositis, is a heterogeneous group of autoimmune diseases usually characterized by chronic inflammation of the muscle with varying clinical manifestations, treatment responses, and prognoses. Epidemiological studies have reported incidence rates for IIM ranging from 2.47 to 7.8 per 100,000 person-years, with prevalence rates ranging from 9.54 to 32.74 per 100,000 individuals (1). IIMs are categorized into subgroups based on clinical and histopathological manifestations, including polymyositis (PM), dermatomyositis (DM, juvenile and adult-onset), amyopathic DM (ADM), and inclusion body myositis (IBM). Recently, the discovery of myositis-specific autoantibodies (MSAs) has led to the identification of new IIM subgroups, such as anti-synthetase syndrome (ASS) and immune-mediated necrotizing myopathy (IMNM), which exhibit distinct clinical phenotypes. Both subgroups and MSAs can aid in the classification of the heterogeneous symptoms of IIM and inform medication and treatment decisions (2–4).

The accurate diagnosis of IIM presents a significant challenge due to the heterogeneity of clinical symptoms and variable responses to immunosuppressive drugs, which are typically used as first-line treatments for autoimmune diseases. There are no standardized therapeutic guidelines for the treatment of IIM due to the lack of robust clinical evidence derived from clinical trials (5). Oral glucocorticoids are typically the first-line treatment for most patients with IIM, while other immunosuppressive drugs and intravenous immunoglobulin may also be used in clinical practice. However, despite these treatment options, some patients may exhibit resistance to therapy, and the underlying reasons for this remain unclear. In recent years, the discovery of MSAs in up to 60% of patients with IIM has represented a significant breakthrough in the field. These MSAs have been proven to be valuable tools in the diagnosis of IIM (6). Autoantibodies such as anti-MDA5, anti-Mi-2, anti-TIF1, anti-SAE, and anti-NXP2 are commonly associated with the DM subgroup. In the ASS subgroup, typical autoantibodies include anti-Jo-1, anti-EJ, and anti-PL7, which target tRNA synthetases. The IMNM subgroup is specifically characterized by the presence of anti-HMGCR or anti-SRP autoantibody. A subset of patients with IIM, approximately 20-30%, exhibit no known autoantibodies and are classified as seronegative IIM (MSA-). MSAs can be associated with clinical manifestations, such as amyopathic myositis and interstitial lung disease (ILD) in anti-MDA5 positive DM patients. However, the underlying mechanisms of different IIM subgroups and MSA groups are still not fully understood (5, 7), and the discovery of new biomarkers is crucial for enhancing our understanding of IIM and improving the accuracy of diagnosis and treatment.

Metabolomics is the study focused on the analysis and characterization of low-molecular-weight molecules and metabolites in cells and biological systems (8). The metabolome serves as a comprehensive measure of the inputs and outputs within biological pathways, providing valuable insights into the pathological changes occurring in various diseases (9). Specifically, the analysis of the serum metabolome unveils the global dynamics of metabolism resulting from physiological or pathological alterations (10). This approach presents researchers with the opportunity to uncover pathogenic mechanisms and identify potential biomarkers, facilitating prompt and precise diagnosis. Such advancements address the requirements for the effective treatment of IIM. However, there are few recent studies about the serum metabolome of IIM. Here, we collected serum samples from a cohort of 144 IIM patients from February to September 2022. Our study represents the largest sample size compared to previous investigations on the serum metabolome of IIM patients and encompasses major IIM subgroups and MSA groups. Our study significantly contributes to the field by providing a better understanding of the underlying pathological mechanisms of IIM and identifying new biomarkers that have the potential to enhance clinical diagnosis and management.

In this study, we utilized matrix-assisted laser desorption ionization time-of-flight mass spectrometry (MALDI-TOF-MS) to investigate the serum metabolome of 144 patients with IIM. We conducted an analysis of differentially expressed peaks among various IIM subgroups or MSA groups. To enhance the characterization of these peaks and identify them as differentially expressed metabolites (DEMs), we utilized liquid chromatography-mass spectrometry (LC-MS) for metabolite identification. Following the identification, we conducted enrichment and pathway analysis on these DEMs. Our analysis revealed that the DM subgroup exhibited decreased activation of the steroid hormone biosynthesis pathway, while the non-MDA5 MSA group displayed increased activation of the arachidonic acid metabolism pathway. These results suggested the heterogeneity of pathological mechanisms, particularly within these two pathways, among different IIM subgroups and MSA groups.

## Materials and methods

### Diagnosis of IIM subgroups and measurement of MSA groups

DM was diagnosed based on the criteria of Bohan and Peter (11, 12). CADM was diagnosed based on the criteria of Sontheimer RD (13). ASS was defined by the presence of an anti-synthetase autoantibody in patients with DM or PM according to the criteria of Bohan and Peter (14). IMNM was defined by the presence of anti-signal recognition particle (SRP) or anti-3-hydroxy-3-methylglutaryl-CoA reductase (HMGCR) autoantibodies in patients with proximal weakness and creatine kinase (CK) elevation as per 2018 European Neuromuscular Centre (ENMC) criteria (15). IBM was defined by the Lloyd et al. criteria (16), although no IBM patients were involved in our study. MSA was measured by standard immunoblotting techniques with the diagnostic kit for antibody profile in autoimmune myositis (YHLO, China).

### Serum samples from patients with IIM

All patients in this study were from the outpatient department of Peking Union Medical College Hospital and received varying recommended doses of glucocorticoid therapy depending on their conditions. Blood samples were collected from patients with IIM at the Department of Rheumatology and Clinical Immunology of Peking Union Medical College Hospital from February to September 2022. After clotting at room temperature for 30 min, the blood samples were centrifuged at 2,000×*g* at 4°C for 5 min, and immediately, the supernatant serum was transferred to 1.5 mL Eppendorf tubes and stored at -80°C for further analysis.

### Sample preparation before MALDI-TOF-MS analysis

5 μL of serum was added to 20 μL of methanol. Then, the mixture was placed on a vortex for 5 min in an ice bath to dissociate. The mixture was diluted to 50% methanol with water and centrifuged at 15,000×*g* and 4°C for 15 min. The supernatant was isolated for testing.

### MALDI-TOF-MS analysis

A 7 mg/mL HCCA matrix (in 60% acetonitrile solution) was mixed with 1:1 sample to 2.5 μL. The mixture was spotted on an MTP BigAnchor 384BC MALDI target plate (Bruker Daltonics). Mass spectrometry analysis was performed on a rapifleX MALDI-TOF mass spectrometer with a Smartbeam 3D laser in reflection negative (RN) mode, controlled by flexControl 4.0 (Bruker Daltonics). Sample measurements were performed with a mass range from 100 to 1,200 m/z at a laser frequency of 10,000 Hz.

### Metabolomics analysis based on MALDI-TOF-MS

Data cleaning was processed by the R package MALDIquant (version 1.22) (17). The functions and parameters were listed: transformIntensity, log2 method was used; smoothIntensity, SavitzkyGolay method and 2 halfWindowSize were used; removeBaseline, SNIP method was used; calibrateIntensity, median method was used; alignSpectra, 20 halfWindowSize, 2 SNR, 0.002 tolerance, and lowess warpingMethod were used; detectPeaks, MAD method, 2 halfWindowSize, and 5 SNR were used. There might be some samples with no signal in fact due to the technique. We checked them out by their small number of peaks and removed the abnormal samples. Only one sample had an abnormal duplicate, and was removed with no impact on our data because we had two duplicates for each sample. Filtered samples were processed the same as described above until detectPeaks. The function averageMassSpectra with a mean method was used to merge the duplicates of each sample. Then, the function detectPeaks with the same parameters was used to detect peaks. The function binPeaks with a tolerance of 0.002 was used to group peaks in different samples. The function filterPeaks with a minFrequency of 0.1 was used to filter peaks only presented in less than 10% of samples. For each peak, samples with a 5 SD deviation from the mean of intensities were considered an outlier and set as a missing value. Missing values were imputed using the k-nearest neighbor (KNN) algorithm by the R package DMwR (version 0.4.1). Principal component analysis (PCA) was performed by R package factoextra (version 1.0.7) and FactoMineR (version 2.7). Partial least squares-discriminant analysis (PLS-DA) was performed by the R package mixOmics (version 6.22.0) (18). Differentially expressed peaks were analyzed with the Wilcox test, and peaks with p<0.05 were retained. Metabolite identification was performed by pairing MALDI-TOF-MS MS1 (major adducts in negative mode include [-H]^-^ and [+Cl]^-^) with the LC-MS metabolite database within 100 ppm. Multiple candidates were allowed in the metabolite identification. Enrichment and pathway analysis was performed by Metaboanalyst (https://www.metaboanalyst.ca/home.xhtml) (19). A pathway overview was drawn with the R package pathview (version 1.36.1) (20).

### LC-MS analysis

Data acquisition was performed using a Q Exactive (Thermo Fisher Scientific, USA). A Waters ACQUITY UPLC BEH C8 column (particle size, 1.7 pm; 100 mm (length) × 2.1 mm (i.d.)) was used for LC separation. Mobile phase A was 40% ethyl cyanide in water, and mobile phase B was 10% ethyl cyanide in isopropanol. Both phases A and B had 0.1% ammonium hydroxide (NH_4_OH) and 0.1% ammonium acetate (NH_4_OAc). The flow rate was 0.25 mL/min, and the gradient was set as follows: 0-1 min, 98% B; 1-5 min, 98% B to 30% B; 5-8 min, 30% B to 0% B; 8-14 min, 0% B; 14-16 min, 0% B to 98% B. The QC samples were prepared by pooling aliquots of several subject samples and injecting every sample after washing and balancing (total 5 samples and 5 QC samples).

The data acquisition was operated in full MS scan mode and ddMS2 scan mode. The source parameters were set as follows: spray voltage, 2,500 V or -2,500 V for positive or negative modes, respectively; capillary temperature, 320°C; ion source, HESI. The resolution for full MS scan mode was set at 70,000, and the AGC target was set at 3e6 for both positive and negative modes. The maximum IT was set at 100 ms. The mass range was set at 100-1,500 Da. For the dd-MS2 scan mode, the MS resolution was set at 17,500, and the AGC target was set at 1e5. The maximum IT was set at 50 ms. The collision energy was set at SNCE 20-30-40%.

### LC-MS metabolomics data processing

The raw data were acquired using Xcalibur (Thermo Fisher Scientific, USA). ProteoWizard (version 3.0.22356) was used to convert raw MS data (.raw) files to the.mzML (for full scan mode) or.mgf format (for ddMS2 mode) (21), and the R package XCMS (version 3.18) was used for peak detection, retention time correction, and peak alignment (22–24). The centWave method was used. The XCMS processing parameters were set as follows: mass accuracy for peak detection = 15 ppm; peak width c= (5, 40); snthresh = 10; and minfrac = 0.5. Metabolite identification was performed using MetDNA2 (http://metdna.zhulab.cn/) (25). The metabolite annotation parameters were set as follows: ionization polarity, positive or negative according to the mode chosen; liquid chromatography mode, HILIC; MS instrument, Thermo Orbitrap; collision energy, SNCE_20_30_40%. Metabolites in level 2 or level 3.1 were kept to establish an LC-MS database. Annotated metabolites were categorized into different levels based on the Metabolomics Standards Initiative (MSI) guidelines (26). Metabolites classified as level 1 were identified using standardized procedures that involved accurate mass determination, retention time analysis, and comparison of MS/MS spectra. These identifications must be performed using the same LC conditions as MetDNA2. Level 2 metabolites were identified based on accurate mass matching and MS/MS similarity. Level 3.1 included the remaining known metabolites annotated by MetDNA2, while level 3.2 consisted of unknown metabolites that could not be matched to the existing database. The database used in our study included a total of 1,144 metabolites, with 234 classified as level 2 and 910 classified as level 3.1.

## Results

### Serum collection and IIM groups

Blood samples were collected from a total of 144 patients with IIM at the Department of Rheumatology and Clinical Immunology of Peking Union Medical College Hospital from February to September 2022. Among the patients, there were 26 cases of ASS, 22 cases of CADM, 89 cases of DM, 2 cases of IMNM, 1 case of JDM, and 7 cases of PM. Additionally, the patients were categorized based on the presence of MSAs as follows: 13 cases of anti-EJ, 1 case of anti-HMGCR, 17 cases of anti-Jo-1, 68 cases of anti-MDA5, 2 cases of anti-Mi-2, 5 cases of anti-NXP2, 9 cases of anti-PL7, 1 case of anti-SAE, 1 case of anti-SRP, 10 cases of anti-TIF1, and 17 cases of MSA- (**Table 1**). Due to the limited number of samples, the IMNM and JDM subgroups were excluded from further analysis among IIM subgroups in our study. Anti-EJ, anti-Jo-1, and anti-PL7, which are autoantibodies targeting tRNA synthetases and commonly found in patients with ASS, were combined into the anti-tSy group for subsequent analysis and discussion (27). Except for patients without known MSA in the MSA- group, the remaining patients mainly included patients with DM, although different MSAs were generally associated with varying clinical manifestations and specific IIM subgroups. Anti-MDA5 was strongly associated with ILD, and the frequency of anti-MDA5 positive DM seemed higher in Chinese patients (7, 28). Therefore, we grouped all anti-MDA5 positive patients into the MDA5 MSA group. Other MSAs, including anti-HMGCR, anti-Mi-2, anti-NXP2, anti-SAE, anti-SRP, and anti-TIF1, were classified into the non-MDA5 group. Supported by MALDI-TOF-MS, we hoped to identify new potential biomarkers of IIM subgroups and reveal the pathological mechanisms of IIM.

**Table 1:** Demographic, subgroup, and MSA characteristics of IIM patients.

Level		ASS	CADM	DM	IMNM	JDM	PM
Number		25	22	87	2	1	7
Age (SD))		51.40	50.32	43.60	33.50	11.00	50.71
		(9.74)	(13.14)	(12.98)	(7.78)	(NA)	(17.47)
Sex (%)	Female	18(72.0)	17 (77.3)	59 (67.8)	0 (0.0)	1 (100.0)	5 (71.4)
	Male	7 (28.0)	5 (22.7)	28 (32.2)	2 (100.0)	0 (0.0)	2 (28.6)
MSA (%)	EJ	5 (20.0)	2 ( 9.1)	5 ( 5.7)	0 (0.0)	0 (0.0)	1 (14.3)
	HMGCR	0 ( 0.0)	0 ( 0.0)	0 ( 0.0)	0 (0.0)	0 (0.0)	1 (14.3)
	Jo-1	14(56.0)	0 ( 0.0)	1 ( 1.1)	0 (0.0)	0 ( 0.0)	1 (14.3)
	MDA5	0 ( 0.0)	13 (59.1)	52 (59.8)	1 ( 50.0)	1 (100.0)	0 ( 0.0)
	Mi-2	0 ( 0.0)	1 ( 4.5)	4 ( 4.6)	0 (0.0)	0 (0.0)	0 ( 0.0)
	MSA-	1 ( 4.0)	4 (18.2)	8 ( 9.2)	1 ( 50.0)	0 (0.0)	3 (42.9)
	NXP2	0 ( 0.0)	0 ( 0.0)	5 ( 5.7)	0 (0.0)	0 (0.0)	0 ( 0.0)
	PL7	5 (20.0)	0 ( 0.0)	4 ( 4.6)	0 (0.0)	0 (0.0)	0 ( 0.0)
	SAE	0 ( 0.0)	0 ( 0.0)	1 ( 1.1)	0 (0.0)	0 (0.0)	0 ( 0.0)
	SRP	0 ( 0.0)	0 ( 0.0)	0 ( 0.0)	0 (0.0)	0 ( 0.0)	1 (14.3)
	TIFl	0 ( 0.0)	2 ( 9.1)	7 ( 8.0)	0 (0.0)	0 (0.0)	0 ( 0.0)

IIM, idiopathic inflammatory myopathies; ASS, anti-synthetase syndrome; CADM, clinically amyopathic dermatomyositis; DM, dermatomyositis; IMNM, immune- mediated necrotizing myopathy; JDM, juvenile dermatomyositis; PM, polymyositis; MSA, myositis-specific antibody; EJ, glycyl-tRNA synthetase; HMGCR, 3-hydroxy-3- methylglutaryl-coenzyme A reductase; Jo-1, histidyl-tRNA synthetase; MDAS, melanoma differentiation-associated gene 5; Mi-2, nucleosome remodeling deacetylase complex; MSA-, MSA negative; NXP2, nuclear matrix protein 2; PL7, threonyl-tRNA synthetase; SAE, small ubiquitin-like modifier activating enzyme; SRP, signal recognition particle; TIF1, transcriptional intermediary factor 1.

### The serum metabolome of IIM was detected by MALDI-TOF/TOF and LC-MS

**Figure 1:**
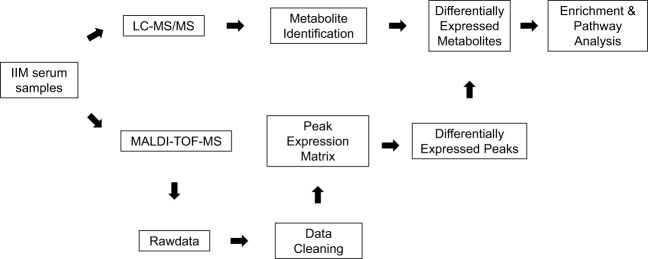
The pipeline for serum metabolome analysis of IIM based on MALDI-TOF-MS. Serum samples from 144 patients with IIM were processed in our study. The peak expression matrix from MALDI-TOF-MS after data cleaning was analyzed by the Wilcox test to filter differentially expressed peaks. These differentially expressed peaks were annotated by the metabolite annotation results from the LC-MS database established by several mixed serum samples of patients with IIM. Enrichment analysis and pathway analysis were performed on Metaboanalyst online.

Serum samples from 144 IIM patients were analyzed by MALDI-TOF-MS and LC-MS by the pipeline shown in **Figure 1**. The raw data obtained from MALDI-TOF-MS underwent a cleaning process to generate a peak expression matrix. Subsequently, the Wilcox test was employed to identify differentially expressed peaks among the IIM subgroups or MSA groups. Metabolite identification of these differentially expressed peaks was supported by utilizing the LC-MS database, which was constructed using several mixed serum samples from IIM patients. Specifically, metabolites annotated as level 2 and level 3.1 by MetDNA2 were retained for establishing the LC-MS database, as described in Materials and Methods. The DEMs annotated by the LC-MS database were subjected to the enrichment and pathway analysis using Metaboanalyst.

### Enrichment analysis of DEMs among IIM subgroups

**Figure 2:**
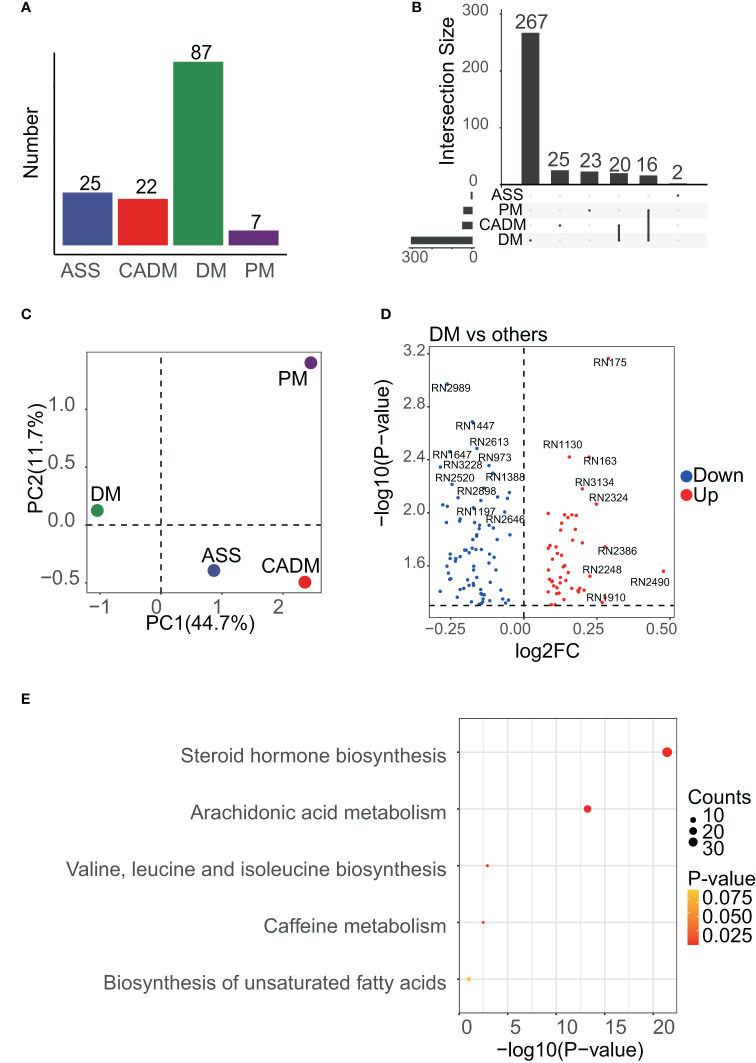
Enrichment results of DEMs among IIM subgroups. **(A)**, the number of samples in each subgroup is shown, and the DM subgroup was dominant. **(B)**, UpSet analysis showed the DEMs among IIM subgroups. DEMs linked between two or more subgroups indicated that these subgroups had common DEMs. The number of these DEMs, unique or common, in each subgroup was labeled in the bar plot. **(C)**, PCA results of IIM subgroups. PC1 meant the first principal component and PC2 meant the second. The mean center of each subgroup was drawn in the plot to represent the subgroup. **(D)**, volcano plot showing the differentially expressed peaks of the DM subgroup versus other subgroups. **(E)**, the enrichment results of DEMs among IIM subgroups.

After performing data cleaning, we obtained a peak expression matrix consisting of 3984 peaks. To investigate the differences among the IIM subgroups, including ASS, CADM, DM, and PM, we conducted comparisons between each subgroup and all other IIM subgroups. Due to the limited number of samples, the IMNM and JDM subgroups were not included in the analysis (**Figure 2A**). Applying a significance threshold of p<0.05, we identified a total of 934 differentially expressed peaks. Among these peaks, 352 DEMs were successfully annotated in 150 differentially expressed peaks. Notably, the DM subgroup exhibited the highest number of DEMs, with 267 unique ones, while the other subgroups demonstrated a substantially smaller number of DEMs (**Figure 2B**). The notable difference in the number of DEMs, particularly with a higher count in the DM subgroup, provides strong evidence of the significant contribution of the DM subgroup to the heterogeneity observed in the serum metabolome of IIM patients. This finding was further supported by the PCA analysis (**Figure 2C**), which clearly shows a distinct separation between the DM subgroup and other subgroups. The differentially expressed peaks were depicted in **Figure 2D**, and the detailed information of annotated metabolites were provided in the **Supplementary Materials**. The results from the enrichment analysis indicated that a majority of the DEMs were associated with the steroid hormone biosynthesis and arachidonic acid metabolism pathways (**Figure 2E**). Additionally, there is moderate involvement of pathways related to amino acid metabolism and biosynthesis of unsaturated fatty acids across the different IIM subgroups.

### Enrichment analysis of DEMs among MSA groups

**Figure 3:**
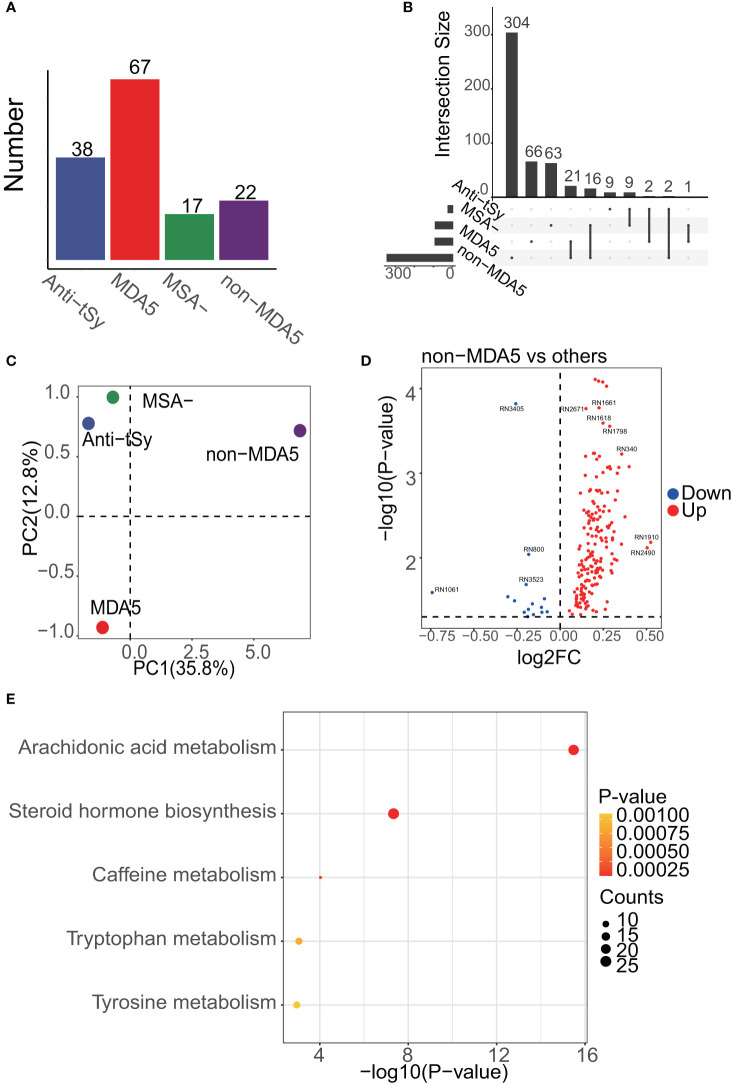
Enrichment results of DEMs among MSA groups. **(A)**, the number of samples in each MSA group is shown, and the MDA5 group was dominant. **(B)**, UpSet analysis showed the DEMs among MSA groups. DEMs linked between two or more MSA groups indicated that these MSA groups had common DEMs. The number of these DEMs, unique or common, in each MSA group was labeled in the bar plot. **(C)**, PCA result of MSA groups. PC1 meant the first principal component and PC2 was the second. The center of each MSA group was drawn. **(D)**, volcano plot showing the differentially expressed peaks of the non-MDA5 group versus other MSA groups. **(E)**, the enrichment results of DEMs among MSA groups.

Each MSA group, namely the anti-tSy, MDA5, MSA-, and non-MDA5 groups, were compared against all other MSA groups as shown in **Figure 3A**. Applying a threshold of p<0.05, we identified 1460 differentially expressed peaks and annotated 491 metabolites with 226 of these differentially expressed peaks. Notably, despite the MDA5 group having the largest number of samples, the majority of the DEMs, including 304 unique ones, were found in the non-MDA5 group (**Figure 3B**). PCA analysis revealed that the non-MDA5 group exhibited relatively distinct differences from the other MSA groups, as depicted in **Figure 3C**. The volcano plot displayed the differentially expressed peaks, while **Supplementary Materials** provided detailed annotation information (**Figure 3D**). Enrichment analysis of DEMs among MSA groups presented similar results to those observed among IIM subgroups. The dominant contributors to the heterogeneity among MSA groups were the steroid hormone biosynthesis pathway and the arachidonic acid metabolism pathway, with the latter ranking first (**Figure 3E**). Additionally, other pathways, including caffeine metabolism, tryptophan metabolism, tyrosine metabolism, valine, leucine and isoleucine biosynthesis, and biosynthesis of unsaturated fatty acids, showed moderate influences among MSA groups.

### Pathway analysis of the DM subgroup and the non-MDA5 group

**Figure 4:**
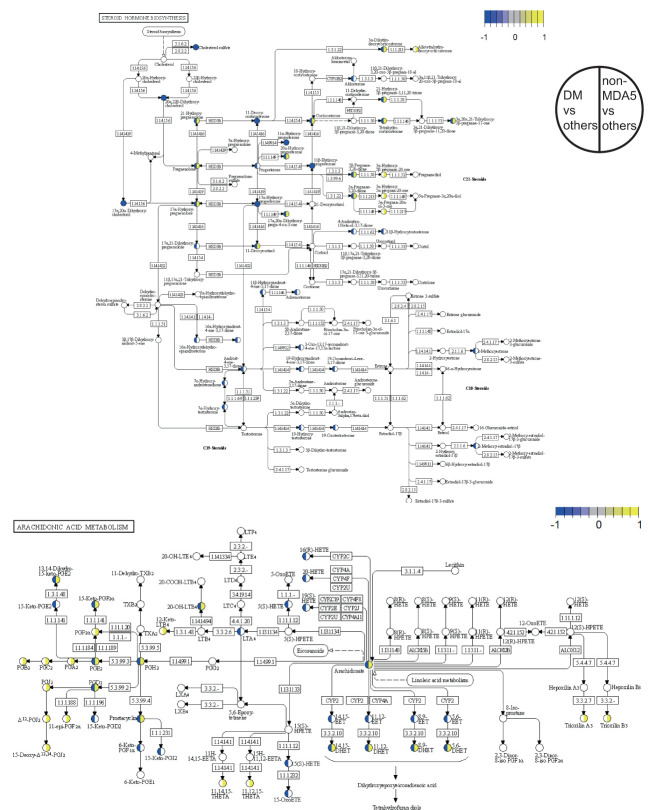
Pathway overview of DEMs in the DM subgroup and non-MDA5 group. The DEMs of the DM subgroup and the non-MDA5 group were marked in the steroid hormone biosynthesis pathway and arachidonic acid metabolism pathway. Metabolites marked in yellow were upregulated against other subgroups or MSA groups, while those marked in blue were downregulated.

A detailed view of the DEMs mapped to the steroid hormone biosynthesis pathway and the arachidonic acid metabolism pathway was presented in **Figure 4**. Within the steroid hormone biosynthesis pathway, the DM subgroup primarily exhibited downregulation of DEMs, indicating a potential disruption in steroid hormone metabolism. The non-MDA5 group displayed upregulation of certain DEMs within the steroid hormone biosynthesis pathway. Similarly, most DEMs of the non-MDA5 group in the arachidonic acid metabolism pathway were upregulated, and only some of them were downregulated in the DM subgroup. Notably, several downstream metabolites, such as prostanoids and dihydroxy eicosatetraenoic acids, were significantly upregulated in the non-MDA5 group, indicating their potential involvement in the arachidonic acid metabolism pathway.

### Typical DEMs in the DM subgroup and the non-MDA5 group

**Figure 5:**
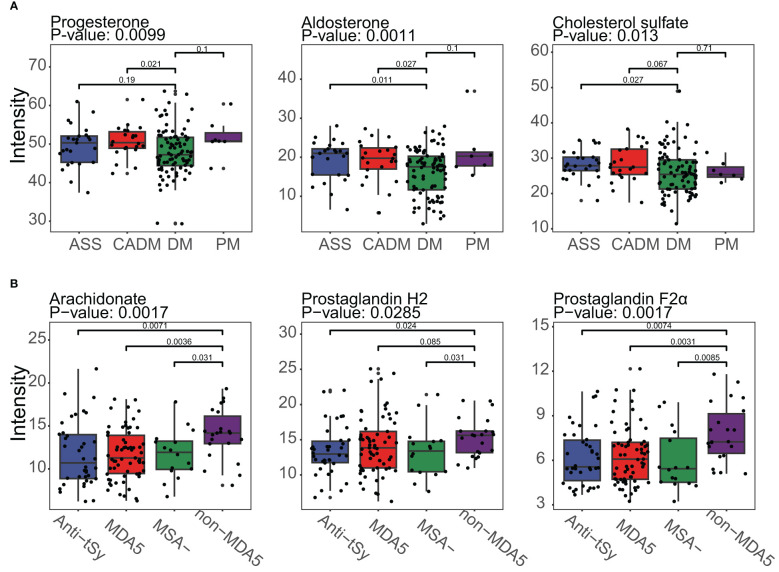
Boxplot of typical DEMs of the DM subgroup and the non-MDA5 group in the steroid hormone biosynthesis pathway and arachidonic acid metabolism pathway. **(A)**, Progesterone, aldosterone and cholesterol sulfate in the steroid hormone biosynthesis pathway were downregulated in the DM subgroup. **(B)**, Arachidonate, prostaglandin H2, and prostaglandin F2α were upregulated in the non-MDA5 group.

The identification of metabolites using MS1 data from MALDI-TOF-MS was limited when it came to annotating metabolites with the same mass. This might result in potential mistakes in overestimation of the impact of certain DEMs within a pathway, as a single differentially expressed peak could represent multiple metabolites. Unfortunately, no suitable solution currently existed to overcome these challenges. However, it was worth noting that despite the limitations mentioned earlier, there were specific metabolites that could be individually annotated by a single differentially expressed peak. Examples of such metabolites included aldosterone, cholesterol sulfate, and arachidonate (as shown in **Figure 5**). These metabolites held particular significance as they were considered typical and core components of the steroid hormone biosynthesis pathway or arachidonic acid metabolism pathway. Furthermore, the observed significant downregulation or upregulation of these metabolites in the DM subgroup or the non-MDA5 group adds to the reliability and validity of our study. Notably, within the DM subgroup, the metabolite progesterone (including candidates such as 9,10-DHOME, 12,13-DHOME, 9,10-epoxy-18-hydroxystearate, and (8E,10S)-10-hydroperoxyoctadeca-8-enoate) exhibited downregulation. Conversely, in the non-MDA5 group, metabolites such as prostaglandin H2 (including candidates such as prostaglandin E2, prostaglandin D2, prostaglandin I2, (5Z)-(15S)-11α-hydroxy-9,15-dioxoprostanoate, 20-OH-leukotriene B4, 15-keto-prostaglandin F2α, and 9,10-dihydroxystearate) and prostaglandin F2α (including candidates such as 11-epi-prostaglandin F2α, 11,12,15-THETA, trioxilin A3, trioxilin B3, and 11,14,15-THETA) were found to be upregulated.

### Model to predict DM subgroup and non-MDA5 group

**Figure 6:**
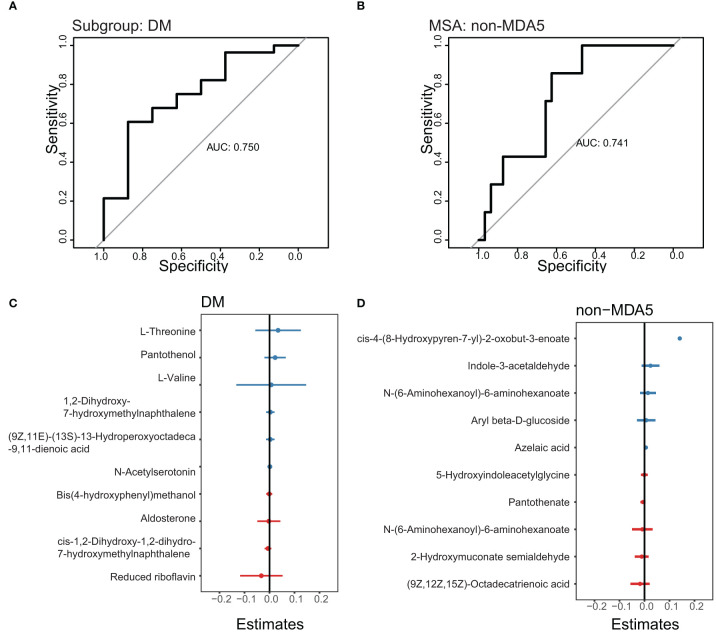
ROC curve to predict the DM subgroup and non-MDA5 group. The prediction models of the DM subgroup and non-MDA5 group. **(A)**, ROC curve to predict the DM subgroup. **(B)**, ROC curve to predict the non-MDA5 group. **(C)**, the effect of metabolites on the DM prediction model. **(D)**, the effect of metabolites on the non-MDA5 prediction model.

To develop prediction models for specific IIM subgroups or MSA groups, we utilized the top DEMs annotated by the LC-MS database between the DM subgroup and other subgroups, as well as between the non-MDA5 group and other MSA groups. These DEMs were used as features in a generalized linear model. The performance of these prediction models was evaluated using receiver operating characteristic (ROC) curves, as shown in **Figure 6**. We focused on the DM subgroup and non-MDA5 group due to their larger number of DEMs. Given the limited number of samples available in our study, the results obtained from the generalized linear model were dependent on the sampling of the training set and test set. To ensure transparency and reproducibility, we provided detailed information regarding the composition of the training set, test set, and the specific DEMs chosen as features for the prediction models in the **Supplementary Materials**. Despite the inherent variability associated with the small sample size, our model demonstrated promising predictive performance. Multiple repeats of the sampling process yielded an average AUC of approximately 0.7. This indicates that our model exhibited a moderate level of predictive ability, suggesting its potential for aiding in the identification and classification of specific IIM subgroups and MSA groups.

## Discussion

In our study, we observed significant downregulation of certain metabolites associated with the steroid hormone biosynthesis pathway in the DM subgroup. Notably, progesterone, a metabolite derived from cholesterol, showed decreased levels in the DM subgroup. Progesterone is known to have anti-inflammatory properties and is involved in regulating both innate and adaptive immune responses. Its role in suppressing inflammation is crucial for maintaining immune homeostasis. Cholesterol sulfate is known to have a significant impact on the regulation of inflammation by modulating key targets. One of its crucial roles is the suppression of leukotriene biosynthesis, a process involved in inflammatory responses. Leukotrienes are inflammatory mediators derived from arachidonic acid metabolism. The enzyme 5-lipoxygenase facilitates the conversion of arachidonic acid into leukotrienes, but this conversion specifically occurs on the nuclear membrane. Cholesterol sulfate acts by reducing the interaction between 5-lipoxygenase and the nuclear membrane, thereby inhibiting the production of leukotrienes (29). Aldosterone increases blood pressure by signaling the kidney and colon to release sodium into the bloodstream. Studies conducted on mouse and rat models have reported that aldosterone infusion can impair endothelial function, suggesting a potential negative impact on blood vessel health (30–33). Patients with DM commonly present blood vessel damage in the perimysium, and the lower levels of aldosterone found in the DM subgroup may reflect an adaptive response to the presence of vascular damage.

The upregulation of typical metabolites in the arachidonic acid metabolism pathway observed in the non-MDA5 group is of notable interest. Arachidonate, a key metabolite in this pathway, plays crucial roles in various biological processes such as cardiovascular biology, carcinogenesis, and inflammatory diseases (34). Beyond the previously mentioned conversion of arachidonate to leukotrienes, there are several other important mediators in the arachidonic acid metabolism pathway. Prostanoids are derived from arachidonate and are involved in diverse physiological and pathological processes, including inflammation and vascular homeostasis. Epoxyeicosatrienoic acids (EETs), dihydroxyeicosatetraenoic acid (diHETEs), eicosatetraenoic acids (ETEs), and lipoxins (LXs) are also metabolites within this pathway, and they play significant roles as signaling molecules and regulators of inflammation and vascular function. EETs promote progenitor cell differentiation, proliferation, and migration, in addition to influencing capillary formation inflammation and apoptosis in endothelial cells (34). The enhanced activation of the arachidonic acid metabolism pathway in certain patients with DM may contribute to the observed vessel damage in the perimysium.

Based on our analysis of the serum metabolome in patients with IIM, we observed lower levels of metabolites associated with the steroid hormone biosynthesis pathway in the DM subgroup, while the non-MDA5 group exhibited higher levels of metabolites involved in the arachidonic acid metabolism pathway. IIM is a group of autoimmune diseases characterized by muscle inflammation and damage. However, the exact mechanisms underlying the development and progression of autoimmunity in IIM are not fully understood. It remains unclear whether the immune system directly targets muscle cells, blood vessels, and connective tissue within the muscle tissue itself. Our study had significant implications for the understanding and treatment of IIM. The observed lower levels of steroid hormones and cholesterol sulfate in the DM subgroup suggested that this subgroup might exhibit increased sensitivity to glucocorticoids, one of the first-line treatments for IIM patients. Furthermore, our results suggested that both proinflammatory and anti-inflammatory factors contributed to the manifestation of muscle and skin damage in IIM. However, in the DM subgroup, dysfunction in the anti-inflammatory response played a more prominent role in the development of symptoms, while the higher levels of arachidonate and prostaglandins in the non-MDA5 group indicate an opposite pattern. Targeting and inhibiting the arachidonic acid metabolism pathway could be a more effective approach for anti-inflammatory therapy in the non-MDA5 group.

In our study, we acknowledge the need for further investigation to address certain questions that arose from our findings. One important consideration is the impact of glucocorticoid therapy on the serum metabolome of the patients. Since all patients in our study received varying recommended doses of glucocorticoids based on their individual conditions, the different drug doses and the inherent heterogeneity of patients could potentially interfere with the results. Designing appropriate data adjustments to account for these factors presents a challenge. Additionally, the frequency of anti-MDA5 positive DM patients in our study was found to be higher (59.8%, 52/87) compared to some previous cohort reports in Chinese patients (36.6%, 53/145) (28). The small number of IIM patients in our study and the effect of regions and the hospital selection of patients might contribute to the result. Further investigations involving larger and diverse patient populations are warranted to gain a better understanding of these differences.

Some limitations of our study are listed below. One limitation was the absence of samples from healthy volunteers. This was primarily due to the presence of the batch effect in the MALDI-TOF-MS analysis, which made it challenging to integrate the data from IIM patients and healthy volunteers. We attempted to develop an algorithm to remove the batch effect between plates used in MALDI-TOF-MS, but further experiments and data were needed to refine the approach. Consequently, we decided it was not appropriate to include data from healthy volunteers in our analysis. Due to the inherent variability and complexity of the clinical serum metabolome, achieving significant differences among IIM subgroups or MSA groups suitable for multivariable analysis, such as PCA or PLS-DA, can be challenging. Therefore, we chose the Wilcox test to filter differentially expressed peaks instead of multivariable analysis. Due to the limited number of samples available in certain IIM subgroups or MSA groups, such as IMNM and JDM, our study faced challenges in conducting comprehensive analyses for these specific groups. To address this limitation and ensure reasonable analysis, we made the decision to integrate rare MSA groups into a single group. This integration introduced inherent heterogeneity within the consolidated group. The sample size of patients with IIM in our study was relatively small compared to some large cohort studies, which may have impacted the reliability and generalizability of our findings.

## Data availability statement

The original contributions presented in the study are included in the article/**Supplementary Material**. The data presented in the study are deposited in the National Omics Data Encyclopedia repository (https://www.biosino.org/node/), accession number OEP004162 and OEP004163. Further inquiries can be directed to the corresponding authors.

## Ethics statement

The studies involving human participants were reviewed and approved by the ethics committee of Peking Union Medical College Hospital (JS-2038). Written informed consent to participate in this study was provided by the participants’ legal guardian/next of kin.

## Author contributions

Experiment and data analysis, TH; Sample collection and grouping, XY, JS, and XZ; Advice for data analysis, YHX, ZX, and MC; Advice for clinical diagnosis and disease, XY, JH, FT, JS, and XZ; Writing—original draft preparation, TH; Editing of the manuscript: YX, HC, and JZ; Supervision, WH, QW, and JZ. TH and XY contributed equally to this work and should be considered co-first authors. All authors contributed to the article and approved the submitted version.
